# Metasurface Source Antenna Gain Improvement Using Simple Side Metal Structure

**DOI:** 10.3390/s24206695

**Published:** 2024-10-18

**Authors:** HongGuk Bae, JaeGon Lee, SangWook Park

**Affiliations:** 1 Department of ICT Convergence, Soonchunhyang University, Asan 31538, Republic of Korea; ghdrnr0402@sch.ac.kr; 2 Department of Electronic SW Engineering, Kyungnam University, Changwon 51767, Republic of Korea; jaegonlee@kyungnam.ac.kr; 3 Department of Electronic Engineering, Soonchunhyang University, Asan 31538, Republic of Korea

**Keywords:** metasurface, transmitarray, side metal structure, sidewall, side plate, gain enhancement, patch source

## Abstract

As metasurfaces are in the spotlight, research is being conducted to incorporate them into transmitarray (TA) antennas. Among these, as an attempt to create a low-profile design, a patch antenna classified as low-gain can be utilized as an appropriate source antenna. However, for high efficiency of the TA, the gain of the source antenna must be fundamentally improved. For this, a simple side metal structure was applied to a metallic cross-type slot transmitarray. This acts as a resonant element and reflector by utilizing the electromagnetic wave radiated from the source antenna. The changes in the center frequency and gain due to the application of the side metal structure to the source antenna were analyzed. The gain of the source antenna was improved by a total of 4.63 dB. This is expected to be applied to create various source waves and to conduct future research on improving the gain in transmitarray antennas.

## 1. Introduction

Antennas are a fundamental technology in communication systems, playing a crucial role in applications such as satellites, mobile communications, and autonomous driving. Among the various antenna technologies, TAs have recently emerged as a promising candidate to replace traditional high-gain antennas. This increasing interest in TAs stems from their potential to offer enhanced performance while combining advantages in terms of ease of production and cost efficiency. In 2011, metasurfaces capable of controlling transmitted and reflected electromagnetic wavefronts were introduced [1], drawing significant attention and leading to extensive research [2,3,4,5,6,7]. Consequently, earlier studies on TAs [8,9] has evolved, with substantial efforts focused on integrating planar metasurfaces into transmit antennas [10,11,12,13]. These advancements have fostered the exploration of novel antenna designs that dynamically manipulate electromagnetic waves to achieve the desired beam steering and gain characteristics. Moreover, studies on reconfigurable intelligent surfaces (RIS) have gained momentum, as these surfaces enable real-time reconfiguration of electromagnetic behavior, further enhancing the functionality and adaptability of modern communication systems. Considering these developments, transmitarray technology is expected to be widely applied across various fields in the near future.

TAs were first proposed in earlier studies and have been evolving towards designs that offer high aperture efficiency and a low profile [14,15,16,17,18,19,20,21]. To achieve these characteristics, the design of TAs requires a complex structure, incorporating both the array antenna and a power supply structure at the source antenna. Active TAs have been developed to meet these demands and are designed using components such as phase shifters, PIN diodes, and varactor diodes, which are essential for beam steering [22,23]. However, Active TAs are known to incur losses due to the presence of these active elements [24,25]. To mitigate these losses, Active TAs can reduce phase quantization loss by employing 2-bit phase control instead of 1-bit, although this approach necessitates a more complex structure. Despite these efforts, the complexity and associated losses present ongoing challenges in the design of Active TAs. In contrast, Passive TAs do not incorporate active elements and instead consist of an array of unit cells, typically composed of patterned structures. This simpler configuration allows Passive TAs to avoid many of the losses associated with active components, but it also limits the beam steering angle [26,27,28,29,30]. To overcome this limitation, methods such as varying the array structure or adjusting the physical location of the source antenna have been explored [31]. However, these approaches often require the use of a power supply structure or a relatively bulky motor, making them less suitable for the current research trends in metasurface technology.

Metasurface feed antennas typically utilize horn antennas and patch antennas. While horn antennas are generally classified as medium-gain antennas, they have limitations when applied to metasurfaces. Specifically, the longer distance required from the feed to the metasurface makes it difficult to achieve a low-profile design. In contrast, patch antennas offer a closer feed distance, making them a more suitable alternative for low-profile applications. However, patch antennas are inherently low-gain, necessitating methods to enhance their gain to ensure adequate performance when used with metasurfaces.

Several techniques have been explored to improve the gain of patch antennas. One common approach involves using metallic structures. For example, in study [32], a metal plate was used as a reflector to reduce the side lobes and increase the size of the main lobe, thereby improving the overall gain. In this study, applying two metallic plates to a 4 × 1 patch array achieved a gain of 9.8 dBi. Further optimization, by applying two metallic plates to a 4 × 1 patch array, resulted in a significant gain improvement to 16.8 dBi, showing an overall gain increase of about 7 dBi. This enhancement also enabled better control over the direction of the radiation waves.

Another study [33] explored gain enhancement by applying a radiator structure through stacking to a patch antenna. In this approach, a radiator structure was applied to a single antenna element using a stacked 2 × 2 array of patches. This design was based on the principle of a double H-type slot microstrip patch antenna and utilized a combination of coplanar parasitic radiators and radiating patch structures to create a resonant cavity. This structure helped reduce the Q factor and increased the antenna’s bandwidth, thereby improving performance.

Despite these advancements, using a fundamentally low-gain source antenna, such as a patch antenna, on a metasurface imposes limits on the gain and aperture efficiency of the transmitted wave. To address these limitations, a sidewall structure can be employed. For instance, A PEC (Perfect Electric Conductor) sidewall was used to surround a TA composed of four layers [18]. This configuration resulted in a gain improvement of 1.9 dBi compared to the absence of a sidewall. Additionally, the aperture efficiency was significantly enhanced from 36% to 56%. This approach is based on the principle of using a reflector structure, one of the effective methods for improving the gain of patch antennas.

In studies focused on improving the gain of patch antennas, it is often found that the existing gain is insufficient or that complex designs are needed to achieve the desired performance levels. To effectively use a patch antenna as a source for a TA, it is crucial to develop a structure that not only enhances gain but also allows for precise control over the waveform of the radiated waves. Simplifying the design of both the source antenna and unit cell structures is essential for achieving cost advantages, which is a key focus in metasurface research. To address these challenges, this study proposes a novel gain enhancement method by applying a simple metallic structure to a single patch antenna. In our approach, a TA is designed using a patch antenna with improved gain as the feed source, consisting of four layers of metallic cross-slot unit cells optimized to operate at 9 GHz. This study aims to simplify the source antenna design while enhancing performance, aligning with the goals of cost efficiency and effective metasurface application. The paper is organized into three sections: Section 1 introduces the patch antenna design and gain improvement method; Section 2 discusses the design of the unit cells and the overall TA configuration for 9 GHz operation; and Section 3 presents an analysis of the beam pattern and gain performance of the TA using the enhanced patch antenna. In this study, a patch antenna operating at 9 GHz is designed, and both simulation and actual measurement results of its performance, using a side metal structure to improve gain, are examined. The proposed side metal structure includes a metallic sidewall surrounding the source antenna and a metallic side plate that integrates with the patch antenna to increase the intensity of radiated waves. For the basic analysis of the proposed side metal structure, certain efficiency losses of the antenna are not considered when assessing the gain. This innovative design aims to balance improved performance with structural simplicity, facilitating more effective and cost-efficient metasurface applications.

## 2. Design and Analysis of Side Metal Structure for Metasurfaces

**Figure 1:**
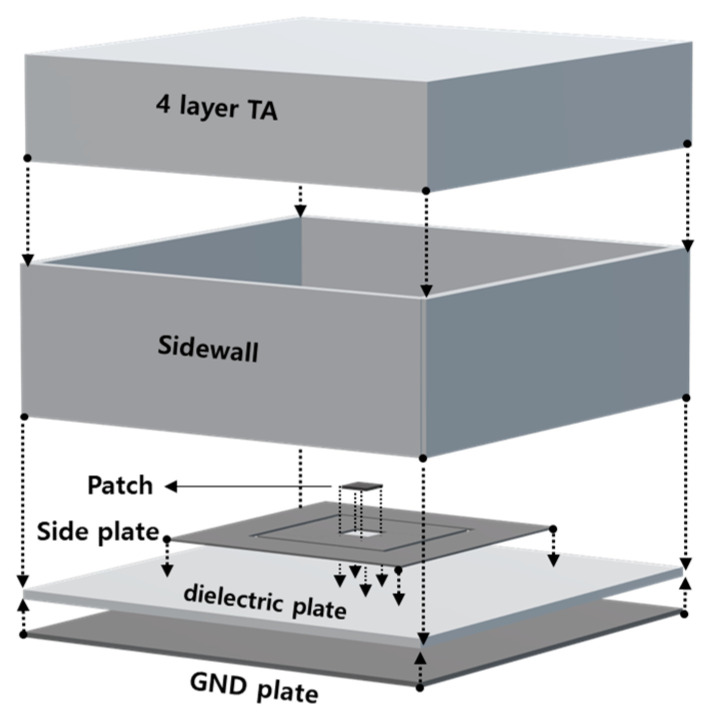
Configuration of proposed TA.

The side metal structure used in this study is composed of two components: the side plate (SP) and the sidewall (SW). As illustrated in Figure 1, the SP is a square ring-shaped structure that is parallel to and surrounds the patch antenna. The proposed TA structure, also shown in Figure 1, consists of a four-layer TA, a source antenna, and an SP implemented using a PCB board, while the SW is made entirely of a copper plate. Section 2 focuses on analyzing the gain variations in the source antenna in relation to different SP configurations and examines the differences between the resonant frequency and the maximum gain frequency, as well as the gain changes resulting from the application of the SW structure. To integrate this design into a metasurface, a cross-type slot TA with a basic structure is developed and applied. The design and analysis of the electromagnetic characteristics are performed Finite Element Method (FEM) electromagnetic simulation with ANSYS HFSS 2021R2.

### 2.1. Patch Source with Side Plate Configuration

**Figure 2:**
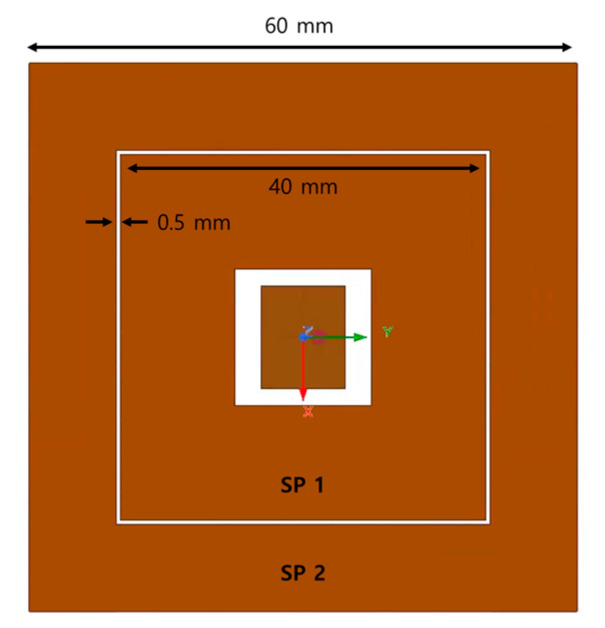
Top view of patch source with side metal plate.

The SP acts as a parasitic coupling element that functions as a resonator by interacting with the electromagnetic field radiated from the patch antenna. This coupling effect is achieved by placing a metal plate around the patch antenna, which serves as a parasitic element to enhance the gain and bandwidth of the antenna [34]. As depicted in Figure 2, two SPs are applied to a single patch, with the patch designed in a square ring shape measuring 9.2 × 11.3 mm, and the metallic side plates sized at 40 × 40 mm and 60 × 60 mm, respectively. The ground plane of the patch source was designed to be 200 × 200 mm to match the width of the TA described in Section 3. The patch antenna was fabricated using a TLX-9 substrate (copper sheet thickness = 0.035 mm, dielectric constant = 2.2, loss tangent = 0.0009), with the side metal plates implemented by printing the copper sheet onto the board like a patch.

**Figure 3:**
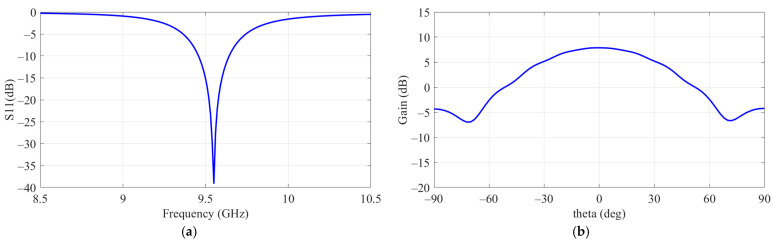
Electromagnetic response of patch source. (**a**) S11. (**b**) Gain (dB).

**Figure 4:**
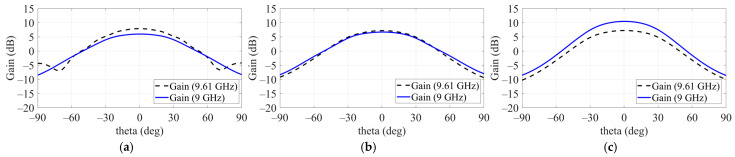
Effect on gain at 9 GHz and 9.61 GHz. (**a**) Patch without SP. (**b**) Patch with one SP. (**c**) Patch with two SPs.

Figure 3 presents the electromagnetic response results of a single patch applied to a ground plane measuring 200 × 200 mm, showing resonance at 9.55 GHz with a gain of 7.8 dB. The simulation results for S11 (dB) and gain (dB) based on the SP structure indicate that applying SP did not affect the resonant frequency. However, when the SW structure was applied, the resonant frequency shifted from 9.55 GHz to 9.61 GHz due to changes in impedance. Despite this shift, the maximum gain was consistently observed at 9 GHz. Figure 4 further illustrates the gain measurements at 9 GHz and 9.61 GHz for a single patch with the SP structure applied but without the SW. The highest gain was recorded when two SP structures surrounded the patch. The simulation results from HFSS show that the optimal configuration for maximum gain was achieved for SP 1 with a side length of 40 mm and SP 2 with a side length of 60 mm.

### 2.2. Patch Source with Sidewall Configuration

The next side metal structure employed in this study is the sidewall, which is made of copper with a thickness of 0.6 mm. As mentioned earlier, the sidewall acts as a reflector, taking advantage of the properties of metal to reflect electromagnetic waves radiated from the antenna. The design of the sidewall requires specifying the focal length, as the height of the sidewall can affect the reflection, beam pattern, and impedance of the electromagnetic waves. Therefore, it is crucial to establish appropriate specifications for optimal performance.

To determine the focal length, [19] standardized the maximum aperture efficiency based on the ratio (F/D), where F represents the distance from the source antenna to the array, and D is the optimal size of the array. For instance, when a patch antenna with a gain of 10 dB is used as the source antenna for a transmission antenna, the maximum aperture efficiency is approximately 62%, and F/D can be designed to be around 0.2. In this study, the maximum gain of the source antenna using the side plate is 10.83 dB, corresponding to an F/D ratio of 0.25. Consequently, the optimal size (D) of the TA, which will be detailed in Section 3, is determined to be 160 mm, with a focal length of 40 mm. Considering the thickness of the patch antenna’s PCB board, the sidewall was designed to have a height of 41 mm.

**Figure 5:**
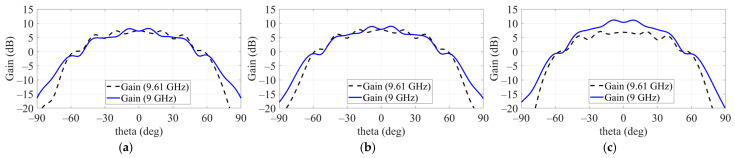
Effect on gain at 9 GHz and 9.61 GHz. (**a**) Single patch without SP. (**b**) Single patch with one SP. (**c**) Single patch with two SPs.

The effect of the sidewall on the source antenna’s gain is shown in Figure 5, where gains were observed at 9 GHz and 9.61 GHz. Comparing (a) and (b), there was no significant change in gain at these frequencies when only a single SP was applied. However, when both SP 1 and SP 2 were applied, the gain improvement was notably larger with the addition of the sidewall. As a result, the overall gain increased from 6.2 dB to 10.83 dB upon applying the side metal structure to the single patch.

**Figure 6:**
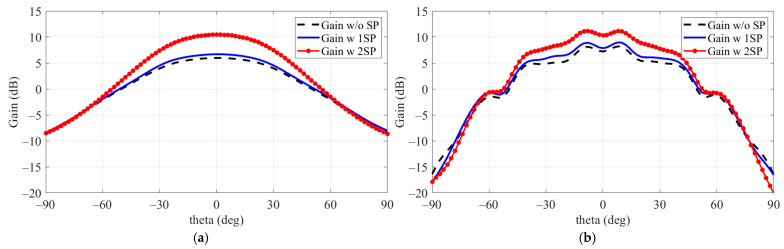
Gain variation in patch antenna at 9 GHz due to SP structure addition, observed before and after SW application. (**a**) Without SW structure. (**b**) With SW structure.

The gain variation in the patch antenna due to the addition of the SP structure was observed for both cases, with and without the application of the SW. As shown in Figure 6, when the SW was not applied, the gain of the patch antenna improved by 4.25 dB, increasing from 6.2 dB to 10.45 dB with the addition of the SP. In the case where the SW was applied, the gain increased by 2.63 dB, from 8.2 dB to 10.83 dB, with the addition of the SP. This improvement highlights the effectiveness of using side metal structures, including both SPs and the SW, in enhancing the gain of the patch antenna.

## 3. Design of Metallic Cross-Type Slot Transmitarray

### 3.1. Design of Metallic Cross-Type Slot Unit Cell

**Figure 7:**
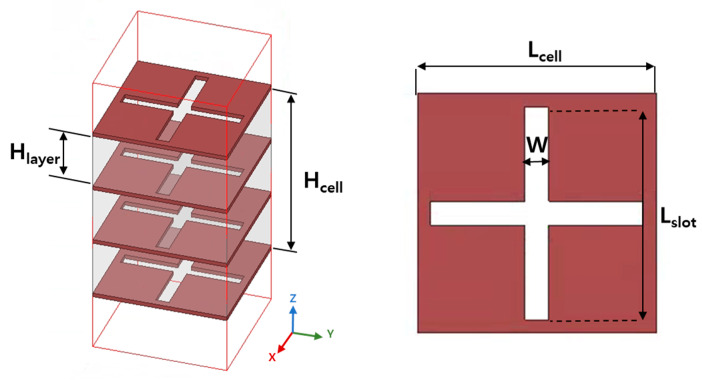
Four-layer metallic cross-type slot unit cell.

In this study, the cross-slot elements that constitute the TA on a PCB board were designed using two approaches. One method involves drilling a cross-slot into a PCB board with both a metal sheet and dielectric structure. The second method involves drilling a cross-slot into a board composed solely of metal. For this study, the latter approach was employed: a cross-slot was drilled into a four-layer unit cell made entirely of copper, as shown in Figure 7. Air was placed between each layer, with no dielectric substrate used. The four layers of the metal plates form a single unit cell, with each plate featuring a cross-type slot.

The copper used for the metal plates had a thickness of 0.6 mm, and the space between the plates was filled with air to allow for an electromagnetic interaction between the slots and the incident waves. The total width of each unit cell was 20 mm (0.6 λ), while the slot width (W) was 2 mm, and the slot length (Lslot) varied to control the transmission phase. By adjusting the length of Lslot, variations in the transmission phase were achieved. This flexibility allows for precise control of the transmission characteristics of the metasurface, as will be necessary when constructing the full unit cell array in the next section.

The source antenna with a metallic side structure demonstrated maximum gain at 9 GHz, so the operating frequency of the metasurface was also targeted at 9 GHz. To match this requirement, the unit cells were fabricated from copper, and the detailed dimensions of the unit cells, including the width (W) of 20 mm (0.6 λ), are listed in Table 1. Each layer of the unit cell was 0.6 mm thick, and the total unit cell width matched the overall TA design.

**Figure 8:**
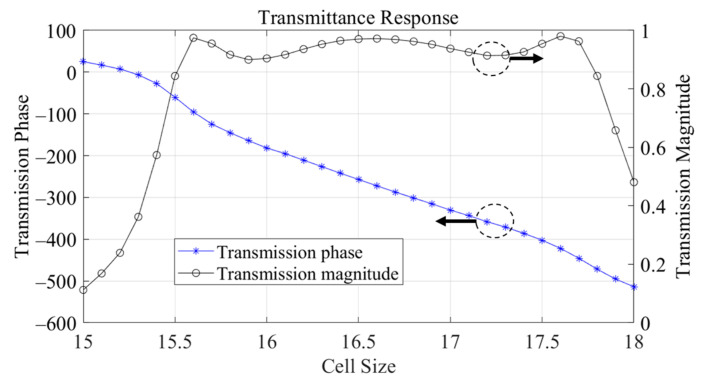
Transmittance response of cross-type metallic unit cell.

For simulation, a Floquet port was applied, with the boundary area set to 0.25 λ of the operating frequency. The transmission phase and transmission magnitude were observed using S-parameters. The transmittance response of the unit cells was obtained at 9 GHz, and as shown in Figure 8, the Lslot values ranged between 15.5 mm and 17.8 mm. The transmission magnitude was greater than 0.8, and a phase range of 412 degrees was achieved.

### 3.2. Design of Proposed Transmitarray Antenna

**Figure 9:**
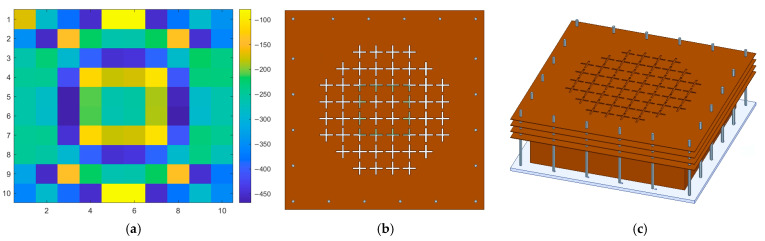
Geometry of TA with 52 elements. (**a**) Phase distribution of TA. (**b**) Top view. (**c**) Sky view of TA with metallic side structure.

For the basic TA experiment, when the beam-steering angle for converting the source wave into a plane wave was set to 0°, the phase distribution result for a 10 × 10 unit cell could be obtained, as shown in Figure 9a. By retaining only the unit cells where the wave radiated from the designed patch antenna reaches the TA, the beam could be focused, thereby improving the aperture efficiency. Additionally, to further enhance the aperture efficiency by reducing the array area, six cells at the corners were removed, forming a circular array with a total of 52 cells, as shown in Figure 9b,c. Additionally, the distance from the substrate—where the patch antenna and SP were designed—to the TA was 43 mm. The previously mentioned F/D ratio was calculated as 0.25, yielding a focal length of 40 mm. However, since the optimal size (D) may not be precisely 160 mm, the height was adjusted within an acceptable range based on the empirical electromagnetic simulation results. It is important to note that as the TA and sidewall are metallic structures, they should not come into contact; thus, a small gap was required.

**Figure 10:**
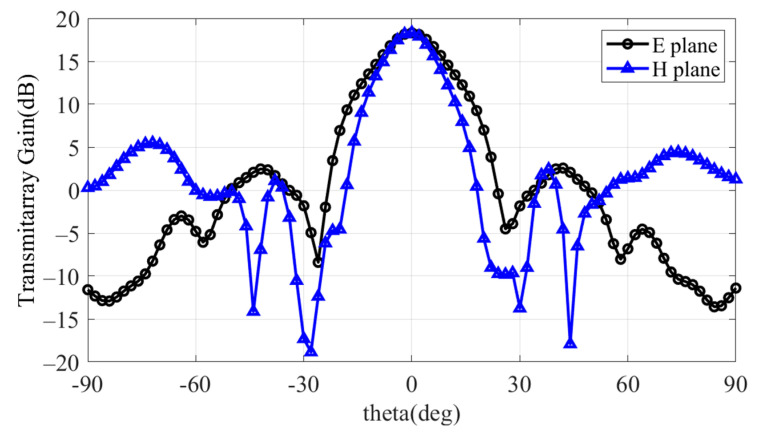
Simulated radiation pattern.

The entire structure was supported by a PVC-type support, which held the four-layer metal plate in place. The designed TA was verified by comparing the simulation results with actual measurements. Since the maximum gain of the source antenna was observed at 9 GHz, the TA achieved a gain of 18.3 dB, resulting in an aperture efficiency of 29.3%, as shown in Figure 10. This was calculated using Equation (1). In this study, the effective area was considered circular, and thus $A_{eff}=\pi r^{2}$ was used for the calculation, with r set to 80 mm.

(1) $$\eta_{a}\left(\%\right)=\frac{G{\lambda_{o}}^{2}}{4\pi A_{eff}}$$

## 4. Experimental Results and Discussion

**Figure 11:**
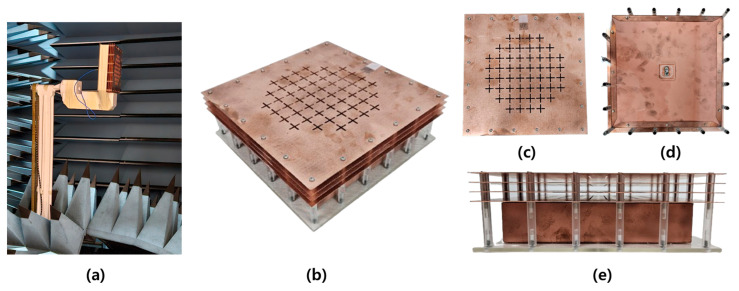
Fabricated prototype. (**a**) Prototype under measurement in chamber. (**b**) Sky view of TA. (**c**) Top view. (**d**) Bottom view. (**e**) Side view.

**Figure 12:**
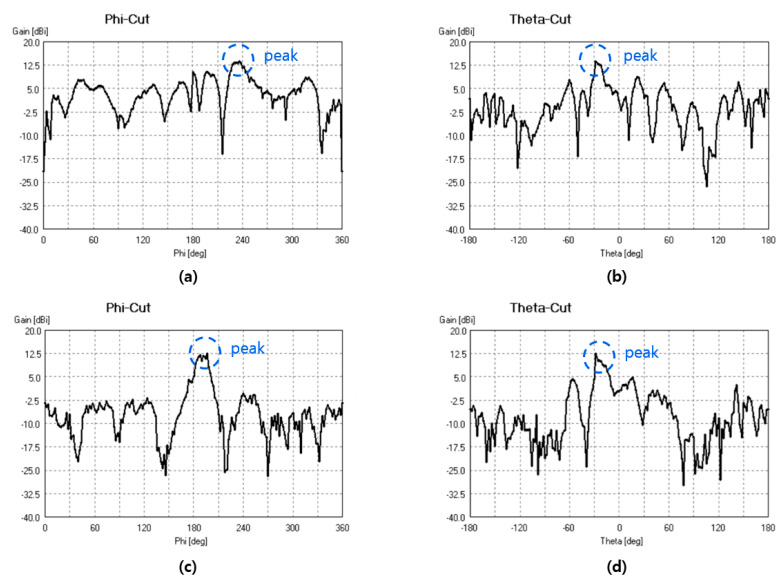
Linear polarization of patch source. (**a**) Phi-cut of theta polarization H. (**b**) Theta-cut of theta polarization H. (**c**) Phi-cut of phi polarization E. (**d**) Theta-cut of phi polarization E.

The performance of the TA designed in this study was evaluated in the chamber depicted in Figure 11. Figure 12 presents the measurement results of the E and H polarizations. The discrepancy between the actual measured gain and the simulated gain is likely due to the insufficient physical rigidity of the support structure holding the antenna within the chamber. During the measurement process, the antenna underwent 360-degree rotation supported by this structure, and it is assumed that measurement errors occurred due to physical deformation of the support structure under this weight.

The actual measurement results revealed a maximum gain of 13.74 dBi for H polarization and 12.58 dBi for E polarization at 9.7 GHz. These maximum values were recorded when Φ was 16 degrees and θ was 26 degrees. While these results suggest that support structure deformation might have contributed to measurement inaccuracies, they cannot solely be attributed to the structure’s deformation due to its heavy weight. Future research will aim to achieve more accurate experimental verification results.

This study proposes a method to enhance the gain of a single patch source by applying a side metal structure to a metasurface. The feasibility of this method was supported by theoretical analysis and simulation results. However, the actual measured gain was approximately 5 dB lower than the simulated gain, indicating the need for further investigation to reconcile these differences.

## 5. Conclusions

In this study, a side metal structure was utilized to improve the gain of the source antenna used in metasurface applications. By employing a simple sidewall (SW) and side plate (SP) structure, the gain of the source antenna was enhanced by 4.63 dB. Electromagnetic simulations were conducted to analyze the gain variations corresponding to each structure. The proposed SP structure was implemented on the substrate used for fabricating the patch antenna, eliminating the need for additional complex structures. A metallic cross-type slot transmitarray was employed to verify the source antenna’s suitability for metasurface applications. Although the experimental results were limited due to constraints in the testing environment, further validation is expected in future studies.

In the study of transmitarray antennas, the profile of the entire structure is greatly affected by the source antenna. In this study, the gain of a patch antenna classified as low-gain was improved to increase its usability as a source antenna of a transmitarray antenna. Improving the gain of patch source antennas for metasurface studies is a significant endeavor, but further study is also essential for generating source waves with diverse beam patterns. Due to the nature of metasurfaces, there are demands in various applications for beam steering, multi-beam generation, and signal enhancement in specific directions. Therefore, continued research on the side metal structure proposed for the source antenna in this study could maximize metasurface performance and expand its applicability to various communication and radar systems.

## Figures and Tables

**Table 1:** Parameters of cross-type metallic unit cell.

Parameter	Value
H_layer_	8.83 mm
H_cell_	27.39 mm
L_cell_	20 mm
L_slot_	15.5~17.8 mm
W	2 mm
Copper plate thickness	0.6 mm

## Data Availability

Data are contained within the article.
